# Effects of rice straw biochar on microbial community structure and metabolic function during anaerobic digestion

**DOI:** 10.1038/s41598-022-10682-2

**Published:** 2022-04-28

**Authors:** Su Wang, Fengmei Shi, Pengfei Li, Fengshan Yang, Zhanjiang Pei, Qiuyue Yu, Xin Zuo, Jie Liu

**Affiliations:** 1 Heilongjiang Academy of Black Soil Conservation and Utilization, Harbin, 150086 China; 2 School of Life Sciences, Heilongjiang University, Harbin, 150080 China; 3 Key Laboratory of Energy Utilization of Main Crop Stalk Resources, Harbin, 150086 China

**Keywords:** Microbiology, Environmental sciences

## Abstract

Anaerobic digestion technology mitigates agricultural organic waste pollution, thereby alleviating the energy crisis. Biochar materials increase the utilisation rate of biomass resources and promote the enrichment and growth of microorganisms. Biochar is an effective exogenous additive that stabilises the anaerobic digestion, improves anaerobic digestion efficiency and gas production. Herein, biochar materials were prepared from rice straw utilising the sequencing batch anaerobic digestion process. The biochar microstructure was characterised by scanning electron microscopy (SEM) and Brunauer–Emmett–Teller (BET) analysis, and microbial succession and metabolic pathways were analysed using 16S rRNA sequencing to reveal the molecular mechanisms. Rice straw biochar addition increased gas production during anaerobic fermentation. SEM revealed that numerous cocci and microbacteria became agglomerated and attached to the surface and pores of biochar, which was revealed by BET analysis to be a good habitat for microorganisms. After anaerobic digestion, the specific surface area and total pore volume of biochar decreased. 16S rRNA gene sequencing showed that biochar affected the abundance of certain bacteria and archaea. Biochar had no obvious effect on the function of bacterial flora but inhibited carbohydrate metabolism by bacteria and glycan biosynthesis and metabolism by archaea in the anaerobic fermentation system while promoting lipid metabolism by archaea. Biochar addition inhibited acetic acid production in the anaerobic fermentation system and promoted methane production based on hydrogen and carbon dioxide levels.

## Introduction

Biochar is a carbonaceous solid residue produced by thermochemical treatments, such as gasification and pyrolysis of biomass materials under anoxic conditions^1^, and has the characteristics of low solubility, high specific surface area, complex pore structure, abundant surface-active groups and strong electrical conductivity^2^. Biochar has been widely studied and applied in the field of soil remediation and water treatment. It can significantly improve the symbiotic relationship of soil microbial communities and increase the activity of microorganisms^3–5^. It is rich in environmentally persistent free radicals, which can activate hydrogen peroxide and induce dissolved oxygen to generate reactive oxygen species^6^, thereby effectively removing or degrading heavy metals and organic pollutants in soil fertilisers and sewage^7,8^. Anaerobic digestion is an effective method to treat livestock manure^9^ and is an important zero-carbon technology that combats global warming and drives the global biogeochemical cycle. Several researchers have found that biochar addition accelerates sludge hydrolysis, aids inhibitor adsorption, enhances microorganism growth and metabolism and promotes interspecific electron transfer, thereby increasing the methane output of anaerobic digestion^10,11^. Pan et al.^12^ studied the effects of different addition ratios of biochar on anaerobic fermentation of chicken manure and found that biochar effectively increased methane production. The highest cumulative methane production induced by biochar from fruit trees obtained at 550 °C was 294 mL/g volatile solid content (VS), which was 69% higher than that of the control. Xu et al.^13^ studied the effect of adding wheat straw biochar on biogas production using pig manure at a moderate temperature (37 ± 1 °C) and found that compared with pure pig manure treatment, biochar addition could shorten the delay period of anaerobic fermentation, which was evidenced by the biogas and methane production increasing by 77.1–96.1% and 78.1–101.8%, respectively.

Biochar addition into the anaerobic digestion system is considered to provide a stable carrier for microorganisms, which is beneficial for the attachment of microorganisms, as it promotes cell fixation and enrichment, aids growth and reproduction and further improves microorganism diversity in terms of number and population structure^14,15^. Cimon et al.^16^ believe that biochar provides a growth location for microorganisms, which is beneficial to the formation of biofilm and protects acetic-acid–producing bacteria and methanogenic archaea from toxic pollutants. Yang^17^ and Lu^18^ stated that the biochar surface can effectively enrich a large number of methanogenic microbial, such as *Methanobacteria* and *Methanococcus*, increase the conversion rate of propionate to acetate, help methanogenic microbial to bear higher volatile fatty acids (VFAs) and further enhance the activity of microbial during the methanogenic stage. Lee et al.^19^ found that *Gordonia*, *Thauera* and *Geobacter* are the main genera attached to the surface of biochar, which are *actinomycetes*, *β Proteus* and *Proteus deltoid*, respectively. Further, biochar materials can improve the metabolic function of microorganisms in the anaerobic digestion system, promote cell growth and cell activity, accelerate microbial metabolism and promote the anaerobic digestion process by providing nutrient elements. Ma et al.^20^ noted that biochar can effectively destroy the structure of insoluble substances in the anaerobic digestion system; increase the contents of total carbon, dissolved organic carbon and inorganic carbon; and accelerate the hydrolysis reaction rate. Duan et al.^21^ found that the hydrolysis efficiencies of proteins, polysaccharides and fats in a reaction system with biochar were 1.4, 1.2 and 1.4 times those in a reaction system without biochar, respectively. Li et al.^22^ found that when biochar is added to the anaerobic digestion system of kitchen waste, the hydrogen-nutrient methanogenic process is the main mechanism of methane production from VFAs at a high temperature, which can promote the material and energy exchange process between mutually operating oxidising bacteria and methanogenic bacteria.

At present, there are numerous related studies on the influence of biochar materials prepared from algae, livestock manure, fruit trees, rice husks, wheat or corn stalks on the anaerobic digestion system^23,24^. Despite the abundant studies on the types and diversity of microbial flora and microbial distribution, the mechanisms underlying the relationship between microorganisms and metabolic functions need to be explored further^23^. Northeast China is an important agricultural area, with a wide rice planting area and well-developed animal husbandry. Animal manure has been well treated by anaerobic digestion, whereas several rice straw resources require an effective, harmless and resource-based treatment to solve the problems of regional ecological environment and resource utilisation. Herein, rice straw was used to prepare biochar material, and its influence on the anaerobic digestion process of livestock and poultry manure was studied. Through characterisation, observation and analysis of microbial flora, the influence of biochar on flora and metabolic function in the anaerobic digestion system was expounded to reveal the mechanism by which rice straw biochar (ZCS) enhances anaerobic digestion and provide a theoretical basis for improving the anaerobic digestion technology of livestock and poultry manure and the practical application of biochar.

## Methods

### Experimental materials

Cow dung was collected from the dairy farm of Miteli Agricultural Development Co., Ltd., Shuangcheng District, Harbin, Heilongjiang Province, China. It was sealed in a transparent polyethylene bag and stored at 4 °C for later use. Inoculated biogas slurry was obtained from the biogas fermenter of a dairy farm. Before the experiment, the inoculated biogas slurry was placed on a shaking table at 35 °C for 2 h and shaken for 24 h to reduce the influence of endogenous biogas on the results. Biochar was prepared from rice straw obtained from the National Agricultural Demonstration Park of Heilongjiang Academy of Agricultural Sciences, Harbin, Heilongjiang Province, China. The rice straw was smashed and sieved through a 40-mesh sieve, then put in a biomass tube furnace(QSK3-9–12, China) at 600 °C for 20 min and subsequently transferred to the air outlet of the carbonisation furnace for 5 min to prevent the high-temperature carbonised products from undergoing spontaneous combustion^10^. After the biochar was cooled to room temperature, it was ground and screened, washed with deionised water to remove ash and dried in an oven at 100 °C for later use. Physical and chemical properties of the test materials are presented in Table 1.

**Table 1:** Basic properties of raw materials.

Material	Dry matter content (TS, %)	Volatile solid content (VS, %)	pH
Cow dung	24.5	96.1	7.7
Inoculate biogas slurry	3.6	1.4	8.4
Rice straw biochar	94.4	83.6	8.2

### Test methods

Biochar derived from rice straw was used in this study and the details of preparation were described in previous studies^10,25^. Through single-factor experimental analysis and response surface experimental design, the optimal technological conditions for the anaerobic digestion of cow dung by biochar addition were compared and determined. The anaerobic fermentation temperature was 41 °C, the total solid content (TS) of cow dung was 8.9% and the amount of rice straw charcoal was 7.1 g/L^25^. Two experimental groups were set up: one in which ZCS was added and a blank control group (ZCK) without the addition of biochar. Three replicates of each group were maintained, and anaerobic fermentation was carried out for 35 days.

An experimental batch anaerobic digestion reactor was used, and the reaction device was a 2000 mL jar. After adding the materials, 500 mL of biogas slurry was inoculated, and the reaction system volume was brought to 1600 mL with distilled water. During inoculation, an anaerobic bottle was filled with 500 mL/min nitrogen gas and maintained for more than 6 min so that the reaction system was maintained in a strict anaerobic environment. The fermentation bottle, gas collection bottle and metering bottle were connected by a latex tube, and the sealing port of the device was sealed with paraffin wax. Before use, air leakage was assessed to ensure tightness of the device.

### Analysis method

#### Determination of physical and chemical indices

To determine physical and chemical indices, sampling should be performed once every 5 days. The pH value was measured using a pH meter (PHS-3G, China). Biochar pH value detection: biochar and ultrapure water were mixed in a mass volume ratio of 1:10 and shaken in an oscillator for 4 h, following which the pH of the supernatant was measured using a pH meter^12^; methane output was measured by draining saturated saline solution^25^; TS was determined by the drying method^13^; VS was determined by the burning method^13^; and ammonia–nitrogen concentration was determined using a multi-wavelength ultraviolet–visible spectrophotometer (760CRT Shanghai, China). Chemical oxygen demand (COD) was measured using a COD analyser (Thermo Orion, USA). VFA concentration was measured using a gas chromatograph (Agilent 7860A, USA).

#### Energy input–output calculation

Energy input–output calculation. The energy consumed by preparing biochar raw materials with unit mass of rice straw and the increase of biogas production efficiency of anaerobic digestion system by adding biochar with rice straw were calculated by calorific value conversion. The energy output ratio was calculated. Among it, the calorific value of methane was calculated according to 34,000 kJ/m^3^. The energy input for biochar preparation was calculated according to formula (1):

1 $$Q = \frac{{P \times (t_{1} + t_{2} ) \times 3600kJ}}{M}$$

where, *Q* is the calorific value consumed by biochar preparation, kJ/g; *P* is the rated power of biochar preparation reaction device, kW; *t*_1_ is the time when the reaction device is heated to the biochar preparation temperature, h; *t*_2_ is the carbonization time after the reaction device reaches the biochar preparation temperature, h; 3600 kJ is the coefficient of 1kWh converted calorific value; *M* is the drying quality in the biochar preparation reaction device, g.

#### Structure characterisation and surface adsorption determination of biochar

The prepared ZCS was dried and stored in a bottle container with a sealed cover at 4 °C and was set as CS. After anaerobic fermentation, biochar samples from the ZCS experimental group were extracted, mixed evenly, dried and set as ZCS.

After gold plating the biochar, the surface structure of biochar was observed using ZEISS MERLIN Compact (Carl Zeiss AG, Jena, Germany). The specific surface area and pore size distribution of biochar were determined using a physical adsorption analyser (ASAP 2460 Accelerated Surface Area and Porosimetry System, Micromeritics Instrument Corporation, Norcross, GA, USA) for nitrogen adsorption. The temperature of the analysis tank was 77.3 K, the sample density was 1 g/cm^3^, the equilibrium interval was 20 s, the free space of environment was 27.9015 cm^3^, the free space of analysis was 83.2155 m^3^ and the low-pressure dose was 15 m/g STP.

#### Microbial succession and metabolic pathway determination

Before and after anaerobic fermentation, DNA was extracted in each treatment, and the DNA extracted in each repeated treatment was mixed evenly after the concentration was measured. The sample of feed liquid before anaerobic fermentation was set as ZC; the sample of feed liquid after anaerobic fermentation in the experimental group with ZCS was set as ZCS and the blank control group without biochar was set as ZCK. Using paired-end sequencing, a library of small fragments was constructed for sequencing. Through splicing, filtering, clustering or denoising of reads, species annotation and abundance analyses were conducted. The 16S rDNA amplification selection region was V3-V4. Bacterial sequencing primers were 341F (Sequence F: CCTACGGGNGGCWGCAG) and 805R (Sequence R: GACTACHVGGGTATCTAATCC), and archaea sequencing primers were 304F (Sequence F: CCCTAYGGGGYGCASCAG) and 1000R (Sequence R: GGCCATGCACYWCYTCTC).

### Data analysis

Data were statistically analyzed and plotted using EXCEL 2013, SPSS 23.0 and Origin 8.0.

## Results

### Effects of rice biochar on anaerobic fermentation

#### Effects of rice biochar on biogas yield

**Figure 1:**
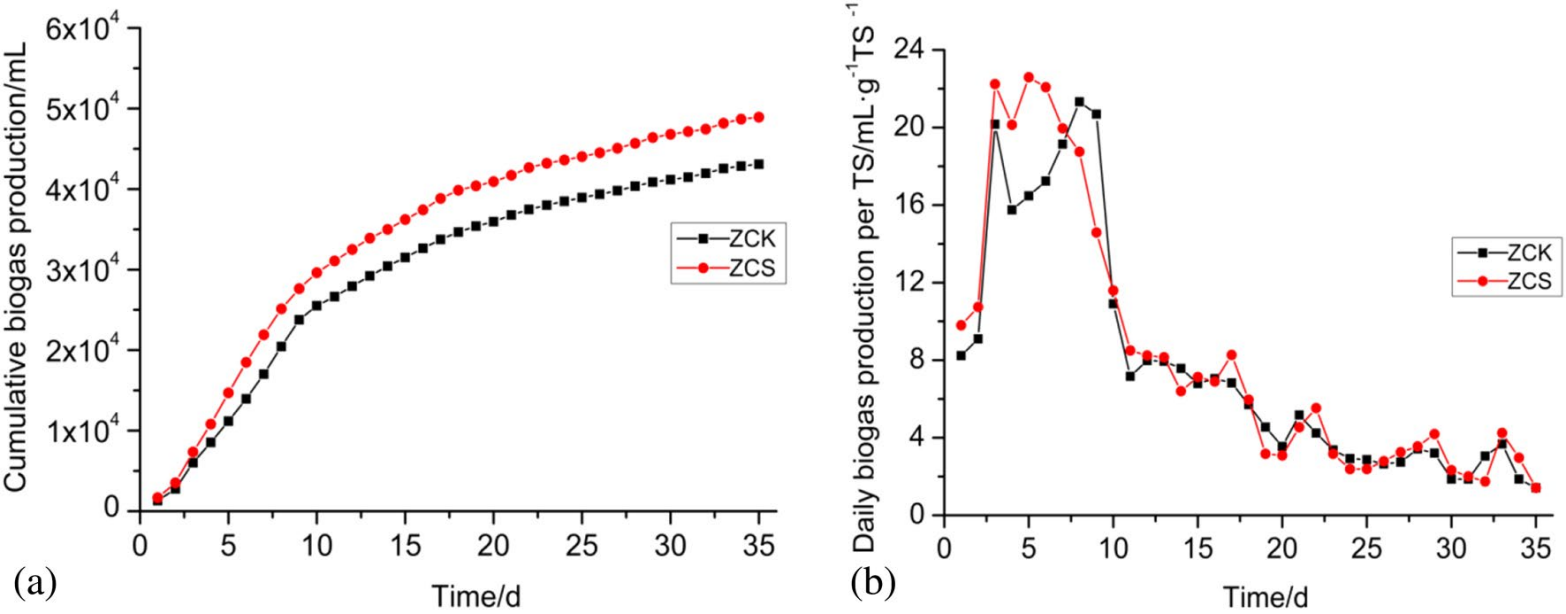
(**a**) Cumulative biogas production; (**b**) Change of biogas production per day.

As shown in Fig. 1a, there was a significant difference between ZCK and ZCS biogas production. On the 35th day after the start of the experiment, the cumulative biogas production of ZCK was 43,077 ± 712 mL, whereas that of ZCS was 48,925 ± 1361 mL, demonstrating a mean increase of 13.58%. As shown in Fig. 1b, the daily biogas yield curve of ZCS showed a rapid upward trend after the start of the experiment, with the biogas yield reaching 22.24 ± 0.65 mL/g·TS^−1^ on the 3rd day and the highest biogas yield reaching 22.59 ± 0.58 mL/g·TS^−1^ on the 5th day. Subsequently, the biogas yield began to gradually decline and reached > 20 mL/g·TS ^− 1^ at the peak of gas production. After 10 days, the gas production rate dropped to < 20 mL/g·TS^−1^, gradually stabilised at 8–5 mL/g·TS^−1^ and entered the final stage of biogas production on the 22nd day. The daily biogas curve of ZCK treatment was similar to that of ZCS treatment, showing a trend of increasing at first and then gradually decreasing. On the 3rd day, the biogas yield reached 20.17 ± 0.31 mL/g·TS^−1^. On the 4th day, the gas production rate obviously decreased and then began to increase. The gas production peak appeared on the 8th day, and the gas production rate was 21.33 ± 0.36 mL/g·TS^−1^. After the 10th day, the daily gas production rate dropped to 4–7 mL/g·TS^−1^. From the gas production conditions of the two experimental groups, it can be seen that the biogas production and daily gas production efficiencies in the ZCS treatment group were significantly higher than those in the ZCK treatment group. The significant analysis was carried out on the changes of ZCK and ZCS daily biogas production, the standard deviation was 366.3188, the *t* value was −2.698, *P* = 0.011, indicating that the addition of biochar had a significant effect on the gas production efficiency of the anaerobic digestion system at the level of < 0.05. Thus, biochar addition can effectively improve the buffering characteristics of the anaerobic digestion system^15^. After the 3rd day of starting the experiment, the biogas production rate of ZCK decreased obviously, which may have been due to the increase of organic acid or ammonia–nitrogen concentration in the reaction system, which caused a certain degree of inhibition.

The rated power of the reaction device is 6 kW in this study. The heating and straw carbonization time is 0.009 h. The amount of biochar prepared from 1 kg of rice straw is 320 g. The calorific value consumed in biochar preparation thought to be an energy input is about 0.6 kJ/g. In terms of energy output, the anaerobic digestion reactor is 1600mLwith the rice straw biochar dosage of 11.36 g. The biogas output was increased by 5818 mL, with methane content of about 55%. According to the calorific value conversion, the productivity improvement after biochar addition is about 9.58 kJ/g, and the input–output ratio is about 1:16.

#### Effects of rice biochar on physical and chemical indices of biogas slurry

**Figure 2:**
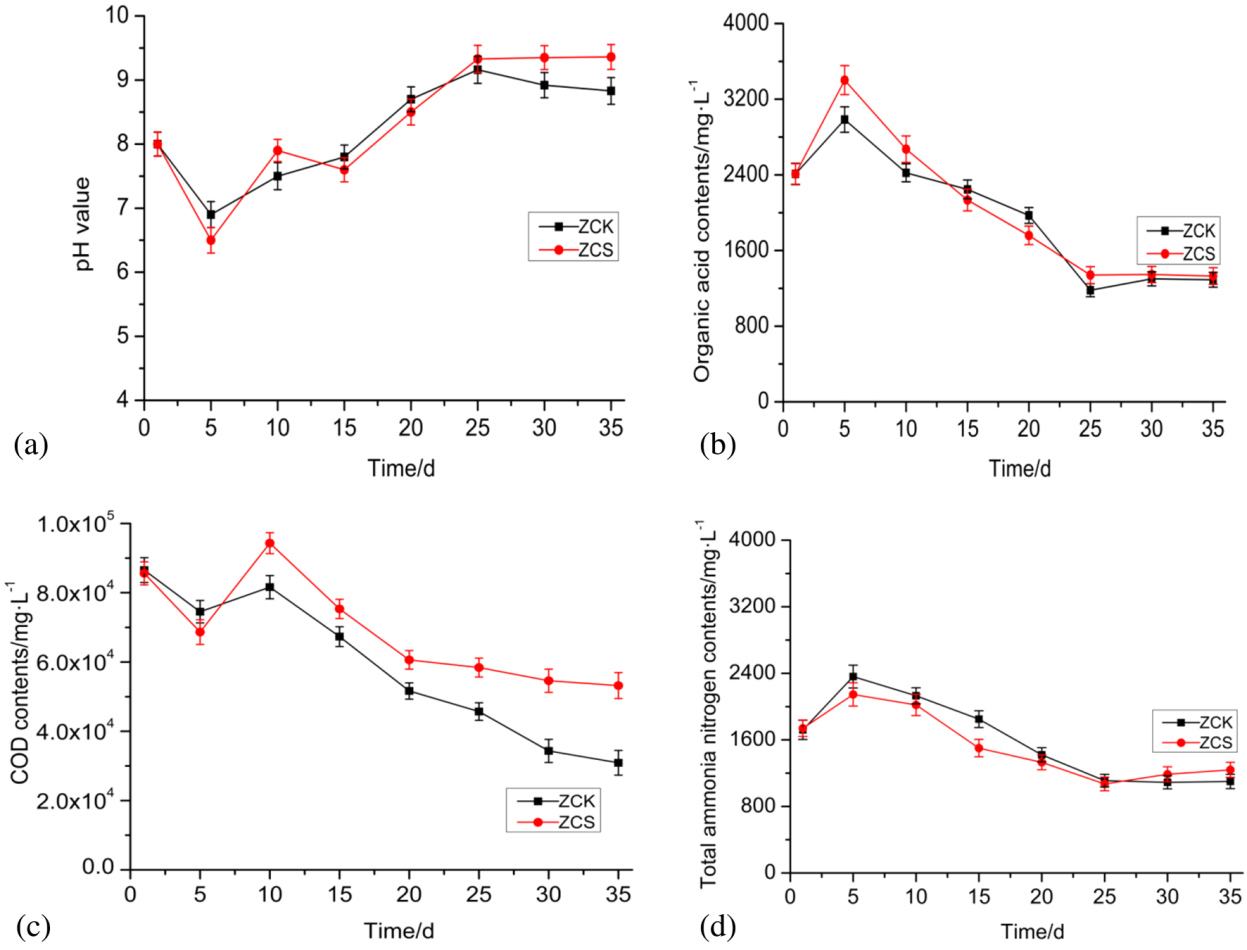
Changes in related physical and chemical indices of the anaerobic digestion system. (**a**) pH; (**b**) organic acid concentration; (**c**) COD concentration; (**d**) ammonia–nitrogen concentration.

The change trends of physical and chemical index curves of the ZCK and ZCS experimental groups were similar, but there was one major difference. As can be seen in Fig. 2a and b, during anaerobic fermentation, the pH value changed from the initial 8. After the start of the experiment, the pH value quickly decreased to 6.5 ± 0.3 and 6.9 ± 0.2, which may have been caused by the transformation and degradation of numerous organic substances into small molecular organic acids. The organic acid content increased from the initial 2410 mg/L to 2985 ± 108 mg/L and 3402 ± 194 mg/L, respectively. However, as methanogenesis progressed, organic acid accumulation gradually decreased. After the 20th day of anaerobic fermentation, the average organic acid content dropped to < 2000 mg/L and gradually stabilised at around 1300 mg/L, whereas the average pH value increased from 7.5 and 7.9 on the 10th day to 9.16 and 9.33 on the 25th day, respectively. The higher pH value may have had a certain inhibitory effect on gas production. Biochar is rich in alkaline-earth metal elements and mostly exists in the form of oxides, which may be the main reason for the increase in the pH value of the anaerobic digestion solution^26^.

As shown in Fig. 2c, the initial COD concentrations of ZCK and ZCS were 8.65 ± 0.36 × 10^4^ mg/L and 8.55 ± 0.32 × 10^4^ mg/L, respectively. After anaerobic digestion began, the organic matter decomposed and the COD concentration dropped rapidly. After the 5th day of anaerobic digestion, a small amount of molecular organic matter gradually accumulated, and the COD concentration gradually increased. On the 10th day, the COD concentration reached the highest level of 9.43 ± 0.33 × 10^4^ mg/L in ZCS. Subsequently, the COD concentrations in ZCK and ZCS began to gradually decrease, but the COD concentration in ZCS was significantly higher than that in ZCK. The COD concentrations in ZCK and ZCS were 3.09 ± 0.42 × 10^4^ mg/L and 5.32 ± 0.49 × 10^4^ mg/L, respectively. As shown in Fig. 2d, with the start of the anaerobic digestion experiment, the ammonia–nitrogen concentrations in ZCK and ZCS rapidly increased from approximately 1700 mg/L to 2361 ± 172 mg/L and 2146 ± 189 mg/L, respectively, on the 5th day and then began to show a gradual downward trend. During the anaerobic digestion process, there was no obvious ammonia–nitrogen inhibition or toxic effect on the methanogenic process^27^. After the 25th day, ammonia–nitrogen concentration in each experimental group was basically stable between 1000 and 1250 mg/L, but the ammonia–nitrogen concentration in ZCS was slightly higher than that in ZCK. On one hand, it may be due to the degradation of more small molecules of organic matter, which released more ammonia–nitrogen; on the other hand, biochar may have adsorbed and degraded ammonia–nitrogen, thereby showing an obvious effect, corroborating the findings of Shi et al.^10^.

### Characterization of rice straw biochar

#### Electron microscopic observation and analysis before and after anaerobic digestion

**Figure 3:**
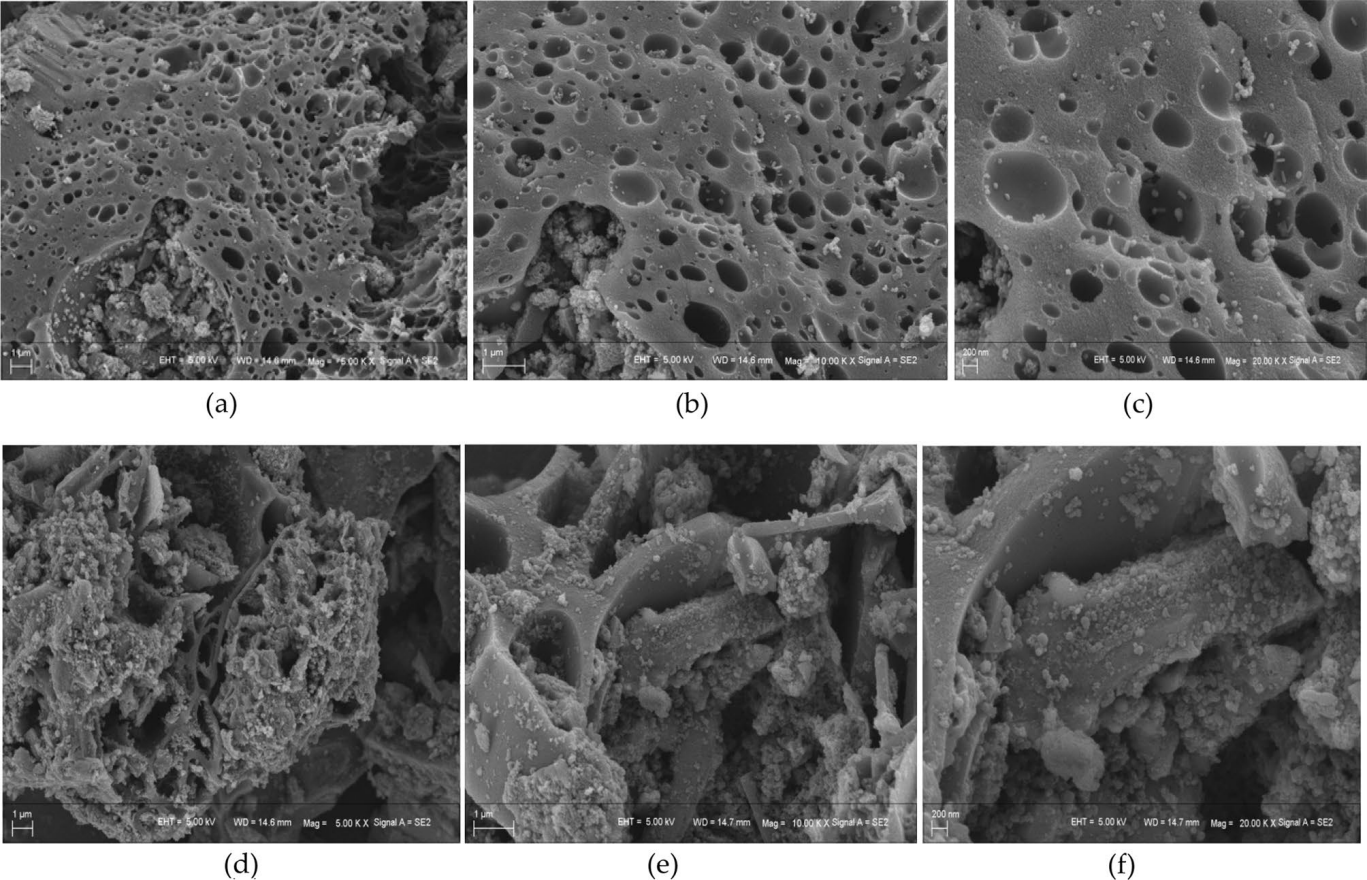
(**a**–**c**) Images of rice straw biochar magnified by 5,000 times, 10,000 times and 20,000 times under a scanning electron microscope, respectively; (**d**–**f**) Images of rice straw biochar after anaerobic digestion magnified by 5000 times, 10,000 times and 20,000 times under a scanning electron microscope, respectively.

The structure of biochar particles before and after anaerobic digestion was observed using scanning electron microscopy (SEM) under the magnification of 5000 times, 10,000 times and 20,000 times. As can be seen in Fig. 3a–c, rice straw biochar had a porous structure, and the surface of microtubules became gradually rougher, which may be because cellulose, hemicellulose and lignin in the straw were decomposed and carbonised. With the support destroyed, the tube wall began to decompose, which caused the biological structure of the biochar to collapse^28^, thereby forming numerous micropores. The pore diameters of some micropores gradually became larger. At the same time, some fine particles accumulated on the surface of biochar, forming rich groups. As shown in Fig. 3d–f, after anaerobic digestion, the porosity and roughness of the biochar material obviously reduced and numerous cocci and microorganisms became attached to and agglomerated around its surface and pores. With its porosity and large specific surface area, biochar can provide a better habitat for microbial growth^29,30^.

#### Brunauer–Emmett–Teller analysis before and after anaerobic digestion

The specific surface area, average pore diameter and total pore volume of ZCS before anaerobic digestion were 57.7016 m^2^/g, 2.7479 nm and 0.0396 cm^3^/g, respectively (Table 2). After adding to the anaerobic fermentation system, the specific surface area and total pore volume decreased to 10.6685 m^2^/g and 0.0187 cm^3^/g, respectively, but the average pore diameter increased to 7.0057 nm. The increase in average pore diameter, specific surface area and total pore volume may be caused by the blockage of the pores of biochar by adsorbed materials. These include microorganisms as well as small particles or colloids in the anaerobic fermentation system. Therefore, it can be seen that biochar anaerobic digestion has good adsorption performance and that the pores of biochar are very likely to become shelter places for microorganisms, which affects the abundance of microorganisms in the anaerobic digestion system^31,32^, consequently affecting the anaerobic efficiency.

**Table 2:** Brunauer–Emmett–Teller analysis of biochar before and after anaerobic digestion.

	Surface area/(m^2^/g)	Adsorption average pore diameter (nm)	Single-point adsorption total pore volume (cm^3^/g)
CS	57.7016	2.7479	0.0396
ZCS	10.6685	7.0057	0.0187

### Microbial study of biogas slurry

#### Analysis of alpha diversity

##### Venn diagrams of microorganisms

**Figure 4:**
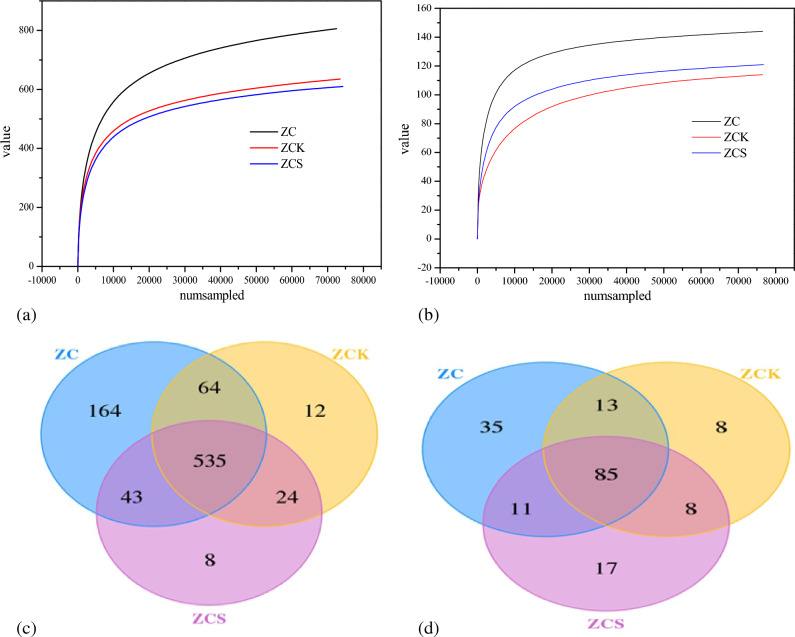
(**a**) Bacterial dilution curve; (**b**) Archaea dilution curve; (**c**) Venn diagram of bacteria; (**d)** Venn diagram of archaea.

Alpha diversity is the analysis of species diversity of a single sample, including the observed species, Chao1, Ace, Shannon, Simpson and coverage indices^33–35^. As shown in Fig. 4a and b, when the number of sample sequences was greater than 10,000, the dilution curve index of bacteria and archaea tended to flatten and all the coverage rates were higher than 0.999, which indicates that all biological populations in the sample were detected and that the detection results truly reflected the actual situation of the biological populations in the sample.

According to Fig. 4c and d, 635 and 610 bacterial operational taxonomic units (OTUs) were detected in the anaerobic fermentation systems without rice straw biochar (ZCK) and with rice straw biochar (ZCS), respectively. There were 559 common OTUs, accounting for 88.03 and 91.64% of total OTUs in the system, respectively. Therefore, although the flora of the two systems were different, the difference was not particularly significant. Among them, 114 and 121 archaea OTUs were detected in the anaerobic fermentation systems without rice straw biochar (ZCK) and with rice straw biochar (ZCS), respectively. There were 93 OTUs in total, accounting for 81.58 and 76.86% of the system, respectively. Therefore, there were no substantial differences between the bacterial flora of the two systems, but there was a significant difference between the archaea.

The Chao1, Shannon and Ace indices of the ZCK bacterial community were higher than those of the ZCS bacterial community (Table 3). According to the changes in the Chao1, Ace and Shannon indices, the ZC sample had the richest bacterial community; that is, the diversity of the bacterial community was the highest in ZC, followed by ZCK (without rice straw biochar). The bacterial diversity in ZCS was the lowest. For archaea, the order of the Chao1, Shannon and Ace indices was ZC > ZCS > ZCK. Accordingly, archaeal species were the most abundant in ZC, followed by ZCS. Archaeal diversity was the lowest in ZCK. That is, archaeal diversity in the anaerobic fermentation inoculum was the highest, which decreased following inoculation in the anaerobic system. The Simpson index of archaea and bacteria in ZCS was the lowest, indicating that biochar addition can increase the relative abundance of dominant flora in the system. The addition of biochar mainly affects the succession of archaea.

**Table 3:** Changes in biodiversity indices.

	Sample ID	Feature	ACE	Chao1	Simpson	Shannon	Coverage
Bacteria	ZC	806	877.8809	895.7581	0.9704	6.7738	0.9985
	ZCK	635	691.4345	722.7778	0.9756	6.7703	0.9989
	ZCS	610	652.5486	659.1333	0.9622	6.3684	0.9991
Archaea	ZC	144	149.1929	155.25	0.8897	4.0262	0.9999
	ZCK	114	120.0228	121.3333	0.8805	3.6611	0.9998
	ZCS	121	126.695	132	0.8678	3.6915	0.9999

After the addition of biochar into the anaerobic system, the pores of biochar can provide a shelter for organisms. At the same time, active sites, such as OH and –COOH, can participate in buffering the pH of the system, affect the amount of ammonia–nitrogen in the system and continuously provide small molecular organic acids for microbial metabolism in the system. For example, the values of pH, COD, ammonia–nitrogen and small molecular organic acids in the system without biochar after 25 days and 35 days of fermentation were 9.16, 4.57 × 10^4^, 1.11 × 10^3^ and 1.18 × 10^3^ and 8.83, 3.09 × 10^4^, 1.10 × 10^3^ and 1.29 × 10^4^ mg/kg, respectively. Except for ammonia–nitrogen, the concentrations of other organic acids, as evidenced by the values of pH, COD and small molecular organic acids, changed the living environment of the flora, and the anaerobic system gradually provided less organic matter, thereby affecting the abundance of the ancient flora. However, after biochar addition, the values of pH, COD, ammonia–nitrogen and small molecular organic acids at the peak of gas production and end of anaerobic process were 9.33, 5.84 × 10^4^, 1.07 × 10^3^ and 1.34 × 10^3^ and 9.36, 5.32 × 10^4^, 1.24 × 10^3^ and 1.33 × 10^4^ mg/kg, respectively. Except for the large change in the ammonia–nitrogen concentration, the values of pH, COD and small molecular organic acids remained relatively stable, which could affect the ammonia–nitrogen concentration in the anaerobic system, forming a good and stable living environment for ancient flora and consequently affecting its abundance.

##### Flora species analysis

**Figure 5:**
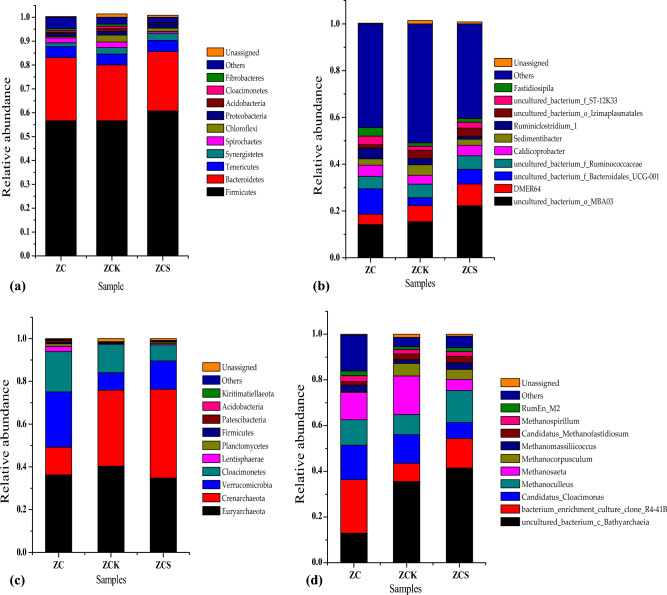
Flora species analysis (**a**) Phyla analysis of bacterial flora species; (**b**) Genera analysis of bacterial flora species; (**c**) Phyla analysis of archaea flora species; (**d**) Genera analysis of archaea flora species.

As shown in Fig. 5a, in the anaerobic fermentation system of the experimental group without biochar (ZCK), there were eight dominant phyla (relative abundance ≥ 1%), namely *Firmicutes* (56.64%), *Bacteroidetes* (23.37%), *Tenericutes* (4.60%), *Synergistetes* (2.67%), *Spirochaetes* (2.341%), *Chloroflexi* (2.95%), *Proteobacteria* (1.533%) and *Acidobacteria* (1.16%). In the anaerobic fermentation system with rice straw biochar (ZCS), there were six dominant phyla (relative abundance ≥ 1%), namely *Firmicutes* (60.72%), *Bacteroidetes* (24.97%), *Tenericutes* (4.64%), *Synergistetes* (3.03%), *Chloroflexi* (1.39%) and *Proteobacteria* (1.57%). *Acidobacteria* (0.18%) and *Spirochaetes* (0.74%) lost their dominant phylum. Therefore, the addition of biochar can increase the relative abundance of *Firmicutes*, *Bacteroidetes*, *Tenericutes*, *Synergistetes* and *Proteobacteria*. Studies have shown that *Firmicutes*, *Bacteroidetes*, *Synergistetes* and *Proteobacteria* are related to the degradation and utilisation of polymeric organic matter, such as lignocellulose, protein and fat^36–38^.

As shown in Fig. 5b, at the bacterial genus level, the relative abundances of *uncultured_bacterium_o_MBA0*, *DMER64*, *Sedimentibacter*, *Ruminiclostridium_1*, *uncultured_bacterium_f_Ruminococcaceae*, *uncultured_bacterium_o_Izimaplasmatales*, *Caldicoprobacter*, *uncultured_bacterium_f_Bacteroidales_UCG-001*, *uncultured_bacterium_f_ST-12K33* and *Fastidiosipila* were among the top 10 in the 2 treatment systems. In ZCS, only the relative abundances of *uncultured_bacterium_o_Izimaplasmatales* and *Caldicoprobacter* decreased by 42.9 and 54.8%, respectively, compared with that of ZCK. The relative abundances of other bacteria increased to varying degrees, and *Sedimentibacter* showed the largest increase, reaching 85.6%, followed by *unguarded_bacteria_f_ST-12k33*, with an increase of 45.1%, and *unguarded_bacteria_o_mba03*, with an increase of 44.2%. *Ruminiclostridium_1* showed the smallest increase (0.26%).

As shown in Fig. 5c, at the level of *archaeophyta*, *Euryarchaeota*, *Crenarchaeota*, *Verrucomicrobia* and *Cloacimonetes* were the dominant phyla in both ZCK and ZCS (relative abundance ≥ 1%). After biochar addition, the relative abundances of *Euryarchaeota* and *Cloacimonetes* decreased from 40.33 and 13.31% to 34.78 and 7.46%, respectively, whereas those of *Crenarchaeota* and *Verrucomicrobia* increased from 23.37 and 8.18% to 41.45 and 13.38%, respectively.

As shown in Fig. 5d, at the level of archaea, the top 10 archaea in terms of relative abundance were *uncultured_bacterium_c_Bathyarchaeia* (12.94–41.45%), *bacterium_enrichment_culture_clone_R4-41B* (7.96–23.35%), *Candidatus_Cloacimonas* (7.05–15.16%), *Methanoculleus*^39^ (8.76–13.98%), *Methanosaeta*^40,41^ (4.84–16.91%), *Methanocorpusculum*^42^ (0.26–5.44%), *Methanomassiliicoccus*^43^ (1.68–2.95%), *Candidatus_Methanofastidiosum* (1.46–2.63%), *Methanospirillum* ^41^ (1.87–2.58%) and *RumEn_M2* (1.26–2.11%). They were all related to the process of methane production. Among them, the relative abundances of the archaea *Candidatus_Cloacimonas*, *Methanosaeta* and *Methanocorpusculum* in ZCS were 43.87, 71.39 and 19.85% lower than that in ZCK, respectively. The abundance of other archaeal genera in ZCS increased, among which that of *Methanomassiliicoccus* increased the most (71.81%), followed by *Bacillus_enrichment_culture_clone_R4-41b* (62.17%), *Methanoculleus* (59.6%) and *Methanospirillum* (8.13%). Therefore, biochar addition can inhibit the metabolic pathway of acetic acid production.

Biochar material has rich specific surface area and hydrophobicity and can be used as an electronic pipeline to provide better electronic conductivity, which can enhance the ability of electronic transmission among microorganisms. According to the change of microbial flora in anaerobic digestion system, the relative abundance of *Methanomassiliicocus* and *Methanoculleus* in ZCS increased by 71.81 and 59.6% than that in ZCK respectively. It is proclaimed that *Methanomassiliicocus* and *Methanoculleus* can secrete hyphae or cytochrome Omcs^44,45^, so that carbon dioxide can be reduced to methane by electron through DIET. In addition, the relative abundance of *Methanospirillum* in ZCS had a relatively small increase of 8.13% than in ZCK. *Methanospirillum* is a hydrogen-trophic methanogenic bacterium. According to some studies^46^, *Methanospirillum* can secrete conductive hyphae and form a symbiotic microorganism with *Syntrophomonas* as an electron donor to form DIET(direct inter-species electron transfer), then promoting anaerobic digestion efficiency. *Methanosaeta* is a typical acetotrophic methanogen and can metabolize acetic acid or ethanol through interspecific electron transfer. However, the relative abundance of methanogens in ZCS decreased greatly by 71.39% than in ZCK. It indicates the methanogenic pathway of acetic acid was severely blocked in ZCS anaerobic digestion system. Therefore, adding rice straw biochar material can effectively improve the abundance of hydrogen-producing trophic methanogenic bacteria in anaerobic digestion system and promote direct inter-species electron transfer.

#### Notes on microbial functions

**Figure 6:**
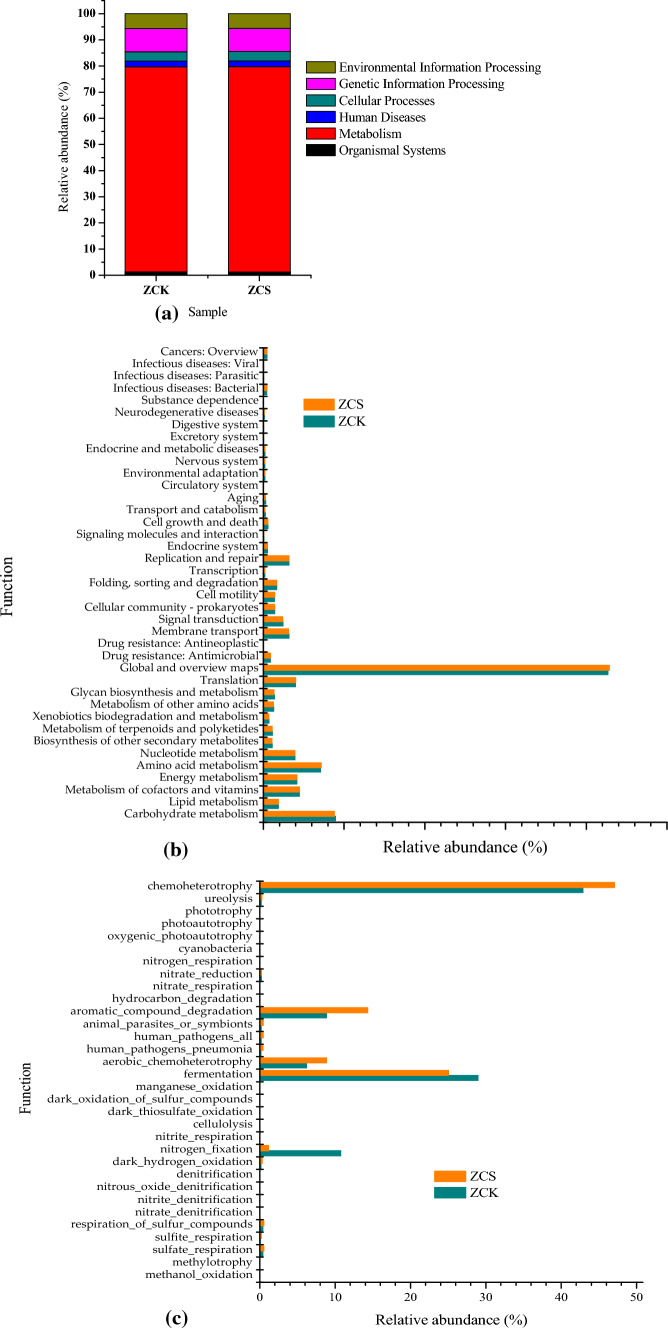
(**a**) Functional notes of bacteria KEGG at level 1; (**b**) Functional notes of bacteria KEGG at level 2; (**c**) Fapotax diagram of bacteria.

Kyoto Encyclopedia of Genes and Genomes (KEGG) data can be used to explore the functions of the bacterial community and main functional proteins^47,48^. As shown in Fig. 6a and b, approximately 78.44–78.50% functions belonged to 12 metabolic pathways in the metabolism group and 5.54–5.64% belonged to 3 metabolic pathways in environmental information processing: membrane transport, signal transduction and signalling molecules and interaction. Further, 1.98–2.03% functional proteins belonged to 11 metabolic pathways in human diseases, 8.92–8.96% were related to 4 metabolic pathways in genetic information processing, 3.56–3.52% of the functions were related to 3 metabolic pathways in cellular processes and 1.25–1.26% of functional proteins belonged to 6 metabolic pathways in organismal systems. Therefore, the effect of rice straw biochar on the function of bacterial flora was not obvious. The relative abundance of carbohydrate metabolism in the metabolic group was the highest in ZCK and ZCS, accounting for 24.9% and 24.6% of the metabolism group, respectively. Therefore, biochar can be said to inhibit carbohydrate metabolism in ZCS.

As shown in Fig. 6c, in ZCK and ZCS, bacterial communities with allelopathy were the most abundant, with relative abundances of 42.89 and 47.08%, respectively, followed by the bacteria involved in anaerobic fermentation, with relative abundances of 28.97 and 25.04%, respectively; 6.19 and 8.84% of bacterial communities required oxidative heterotrophy, and 8.8 and 14.32% of the bacterial community participated in the degradation of aromatic compounds, respectively. As rice biochar contains aromatic compounds, the addition of biochar increases the relative abundance of bacterial communities that degrade and utilize aromatic compounds and their intermediates, such as aromatic compound-degrading flora and chemoheterotrophic bacterial communities. However, the addition of biochar inhibits the metabolic activities of bacteria involved in anaerobic fermentation. The difference in nitrogen-fixing functional bacteria was the most significant among the differences between the two anaerobic systems.

**Figure 7:**
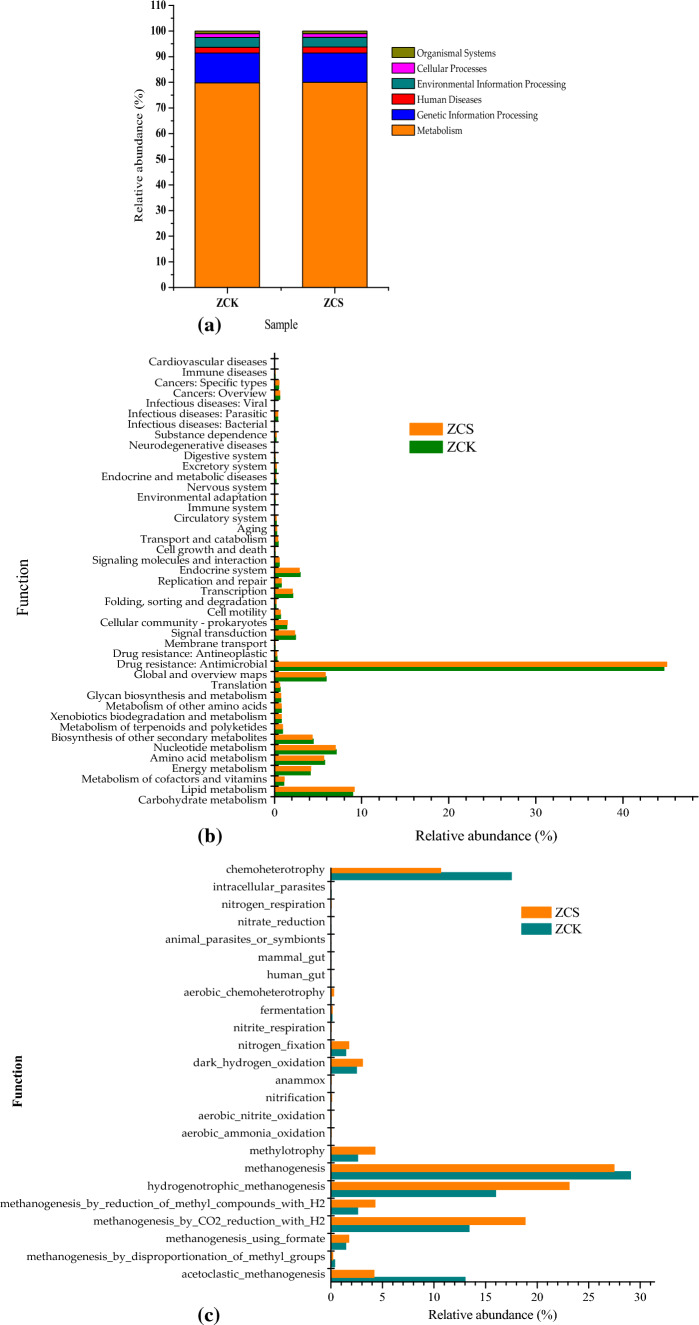
(**a**) Functional notes of archaea KEGG at level 1; (**b**) Functional notes of archaea KEGG at level 2; (**c**) Fapotax diagram of archaea.

KEGG data can be used to explore the functions of the archaeal community and main functional proteins. As shown in Fig. 7a and b, approximately 79.79–80.03% of the functions were related to 12 metabolic pathways in the metabolism group and 3.80–3.87% were related to 3 metabolic pathways in environmental information processing: membrane transport, signal transduction and signalling molecules and interaction. Notably, 2.19–2.29% of functional proteins belonged to 12 metabolic pathways in human diseases, 1.44–11.68% belonged to 4 metabolic pathways in genetic information processing, 1.44–1.45% of the functions were related to 4 metabolic pathways in cellular processes and 1.03–11.03% of the functional proteins were involved in 9 kinds of metabolism in the organismal systems group (KEGG diagram at level 1). The relative abundance of carbohydrate metabolism was highest in the metabolic group in ZCK and ZCS, accounting for 11.2 and 11.4% of the metabolism group, respectively. The relative abundance of genes involved in amino acid metabolism was 7.08 and 6.98%, respectively. After biochar addition, the largest change was in the relative abundance of bacteria involved in glycan biosynthesis and the metabolism pathway, with a decrease of 4.71%, followed by lipid metabolism, which increased by 4.26% compared with the system without biochar addition. Therefore, biochar addition can inhibit glycine biosynthesis and metabolism while promoting lipid metabolism (KEGG diagram at level 2).

As shown in Fig. 7c, in ZCK and ZCS, unlike bacteria, allelopathic archaeal communities were not the most abundant. In ZCK, those with the top five relative abundances were *hydrogenatophic_methanogenesis* (29.06%), *chemohetrophy* (17.5%), *hydrogenatophic_methanogenesis* (15.98%), *methanogenesis_by_CO2_reduction_with_H2* (13.39%) and *acetocratic_methodology* (13.01%). In ZCS, those with the top five relative abundances were *methanogenesis* (27.44%), *hydrogenotrophic_methanogenesis* (23.11%), *methanogenesis_by_CO2_reduction_with_H2* (18.84%), *chemoheterotrophy* (10.66%) and *methodology_by_reduction_of_methyl_compounds_with_H2* (4.27%). In ZCS, the relative abundances of *acetoclastic_methanogenesis*, *methylotrophy*, *methanogenesis_by_reduction_of_methyl_compounds_with_H2*, *chemoheterotrophy*, *methanogenesis_by_CO2_reduction_with_H2*, *hydrogenotrophic_methanogenesis* and *methanogenesis changed* by − 67.93, 64.94, 65.27, − 39.10, 40.62, 44.61 and − 5.57%, respectively, compared with those of similar bacteria in ZCK. Therefore, biochar addition can reduce the relative abundance of bacteria producing methane using acetic acid and increase the abundance of bacteria producing methane using hydrogen, carbon dioxide and methanol as substrates. That is, biochar addition can change the mechanism of methane production in the anaerobic system.

## Discussion

According to research, the addition of rice straw biochar could impact the gas production significiently at 0.05 level (*P* = 0.011). The cumulative gas production of the group with rice straw biochar meanly increased by 13.58% compared with the control group, and the maximum daily gas yield reached 22.59 ± 0.58 mL/g·TS^−1^. In addition, when rice biochar was added into the anaerobic digestion system, the start-up time was not significantly shortened. However, biochar's developed pore structure and rich alkaline-earth metal elements can improve the pH value of anaerobic digestion solution to a certain extent, and have a good effect on the adsorption and degradation of ammonia nitrogen, thus effectively improving the buffering capacity of anaerobic digestion system.

According to SEM and BET analysis, a large number of micropores formed on the surface of rice straw biochar provide a good habitat and shelter for microorganisms, which affected the abundance of microorganisms in anaerobic digestion system. 16S rRNA gene sequencing analysis revealed that the addition of biochar could affect the relative abundance of bacteria and archaea, particularly archaea. Additional enrichment and growth of flora enhance the degradation of organic acids and ammonia–nitrogen in the anaerobic digestion system. The addition of rice straw biochar could increase the relative abundances of *Firmicutes*, *Bacteroidetes*, *Tenericutes*, *Synergistetes* and *Proteobacteria*, which are related to organic degradation. The relative abundance of Sedimentibacter greatly increased, reaching 85.6%. *Euryarchaeota*, *Crenarchaeota*, *Verrucomicrobia* and *Cloacimonetes* were the dominant archaea in ZCK and ZCS anaerobic fermentation systems. The relative abundances of the ancient bacteria *Candidatus_Cloacimonas*, *Methanosaeta* and *Methanocorpusculum* decreased to varied degrees in ZCS compared with ZCK.

Although the addition of rice straw biochar had no obvious effect on the function of bacterial flora, biochar material, which has rich specific surface area and hydrophobicity, can be used as an electronic pipeline to provide better electronic conductivity, thus enhancing the ability of electronic transmission among microorganisms. After adding rice straw biochar, the aceticlastic methanogenesis in anaerobic digestion system was inhibited, and the relative abundance of acetotrophic methanogen such as *Methanosaeta* decreased greatly. On the contrary, the abundance of hydrotrophic methanogen such as *Methanomassiliicocus* and *Methanoculleus* increased significantly, which effectively enhanced the direct inter-species electron transfer. Moerover, the rice straw biochar could inhibit carbohydrate metabolism by bacteria and glycan biosynthesis and metabolism by archaea in the anaerobic fermentation system, promote lipid metabolism by archaea and affect ammonia–nitrogen concentration in the system. Therefore, biochar addition could inhibit acetic acid production in the anaerobic fermentation system and promote methane production based on hydrogen and carbon dioxide levels.

At present, the application of exogenous additives in the anaerobic digestion system has been widely studied. Bentonite, zeolite, magnetite, nano and other materials can promote the attachment of microorganisms^49,50^, the growth and enrichment of microorganisms. The metal elements and mineral salts contained in the materials can also improve the stability of the anaerobic digestion system and the ability of electron conversion^51^. In addition, adsorption materials can be used as carriers to load microbial agents or metal ions^52^, which can further greatly improve the anaerobic performance. In this study, straw biochar was used as an exogenous additive material. Firstly, the raw material of straw biochar is cheap and easy access. Considering the impact on regional ecological environment, most of the biogas residues and biogas slurry wastes produced by biogas production are returned to the field for treatment. Biochar materials have good environmental protection properties, and can be returned to the field with the biogas residues and biogas slurry, without increasing the risk of secondary pollution to the regional environment. Thus, it has a broader application prospect.

The winter is long and cold in northern China, and straw biomass raw materials have been widely used to provide energy for domestic heating in rural areas through technologies such as solidification molding and bundling-based direct combustion. As a result, more biochar can be produced. On the one hand, the recent research at home and abroad indicates that the use of biochar can increase exogenous soil organic carbon, enhance soil carbon fixation capacity and reduce soil bulk density^53^. On the other hand, through this study, the utilization of biochar is further expanded to improve the efficiency of gas production and the energy output benefit of straw biomass utilization. Furthermore, the orderly returning of biogas residue and biogas slurry to the field can not only improve the soil fertility, but also improve soil carbon sink to a certain extent, thus effectively promoting the industrial chain of regional circular low-carbon agriculture development.

In view of the assumption of practical application in biogas engineering, under the situation of continuous feeding and discharging and a large amount of feeding, it can be applied to the anaerobic digestion system with high solid concentration in consideration of ensuring the mass transfer effect and avoiding the precipitation of biochar. It is recommended to adopt commonly used anaerobic reactor such as CSTR and UASB. However, the input of biochar material may increase the material viscosity of slurry in the reactor, and then cause negative influences on slurry flow and stirring intensity in the reaction device. So it is necessary to further explore the engineering process parameters. The biochar is attached to the reaction material, and will also be discharged because of continuous discharge. To better ensure the density of dominant flora in the system, part of biochar can be peeled off from biogas residue on the basis of technological methods exploration and then filled back into the reactor in proportion to ensure the stable and efficient operation of biogas project.
