# Author Correction: Effects of rice straw biochar on microbial community structure and metabolic function during anaerobic digestion

**DOI:** 10.1038/s41598-024-70013-5

**Published:** 2024-08-21

**Authors:** Su Wang, Fengmei Shi, Pengfei Li, Fengshan Yang, Zhanjiang Pei, Qiuyue Yu, Xin Zuo, Jie Liu

**Affiliations:** 1 Heilongjiang Academy of Black Soil Conservation and Utilization, Harbin, 150086 China; 2 School of Life Sciences, Heilongjiang University, Harbin, 150080 China; 3 Key Laboratory of Energy Utilization of Main Crop Stalk Resources, Harbin, 150086 China

Correction to: *Scientific Reports* 10.1038/s41598-022-10682-2, published online 28 April 2022

The original version of this Article contained errors.

The title of the paper was incorrect.

“Effects of rice straw biochar on methanogenic bacteria and metabolic function in anaerobic digestion”

now reads:

“Effects of rice straw biochar on microbial community structure and metabolic function during anaerobic digestion”

In addition, in the Introduction,

“Cimon et al.^16^ believe that biochar provides a growth location for anaerobic bacteria, which is beneficial to the formation of biofilm and protects acetic-acid–producing bacteria and methanogenic archaea from toxic pollutants.”

now reads:

“Cimon et al.^16^ believe that biochar provides a growth location for microorganisms, which is beneficial to the formation of biofilm and protects acetic-acid–producing bacteria and methanogenic archaea from toxic pollutants.”

And,

 “Yang^17^ and Lu^18^ stated that the biochar surface can effectively enrich a large number of methanogenic bacteria, such as *Methanobacteria* and *Methanococcus*, increase the conversion rate of propionate to acetate, help methanogenic bacteria to bear higher volatile fatty acids (VFAs) and further enhance the activity of bacteria during the methanogenic stage.”

now reads:

“Yang^17^ and Lu^18^ stated that the biochar surface can effectively enrich a large number of methanogenic microbial, such as *Methanobacteria* and *Methanococcus*, increase the conversion rate of propionate to acetate, help methanogenic microbial to bear higher volatile fatty acids (VFAs) and further enhance the activity of microbial during the methanogenic stage.”

Finally, in the Methods section, under the subheading ‘Test methods’,

“Based on previous research, we prepared biochar materials from rice straw, corn straw and rice husk.”

now reads:

“Biochar derived from rice straw was used in this study and the details of preparation were described in previous studies^10,25^.”

The original Article has been corrected.

